# The Book World of Medicine and Science

**Published:** 1898-12-03

**Authors:** 


### The Book World of Medicine and Science.

Diet and Food in Relation to Strength and Power of Endurance. By Alex. Haig, M.A., M.D., F.R.C.P. (London: J. and A. Churchill. 1898. Pp. 86. Price 2s.)

Dr. Haig's writings are always interesting, and this latest work of his is no exception to the rule. Although, as may be expected, the teachings of this little book are in great measure deductions from the results of those experiments of Dr. Haig's about which so much controversy has raged, they are none the less practical and valuable. We have often been impressed by the confidence many general practitioners put in the application of Dr. Haig's views. Men of large experience are accustomed to say, rather crudely perhaps, that they care not a whit for the physiologists' criticisms; the thing works out all right. And so it does. Much of the advice given here by Dr. Haig we can cordially endorse, without expressing any opinion on the capacity for evil of uric acid.

But in one matter we must dissent from Dr. Haig. Dr. Haig condemns the use of all stimulants (and with stimulants he classes tobacco), on the ground that they are but means of "calling up our reserves." Dr. Haig says that they precipitate "physiological bankruptcy." But surely it were foolish for a general to lose a battle for fear of exhausting his reserves, or for a financier to suffer discredit rather than to deplete *his* reserves. So long as we recognise that stimulants do but "call up our reserves," it is right to use them in time of stress. The difficult corner once turned, then comes the time for rest and recuperation. Some very excellent observations are made by Dr. Haig on the right use of vegetable foods. For much of the disfavour into which vegetarianism has fallen is due to the unscientific and crude diets that have been put forward. Vegetarianism, when it does not mean proteid starvation and carbohydrate gluttony, is a valuable weapon in the hands of a wise physician. On the whole, Dr. Haig's book is well worthy of consideration. Though, as we have said, it is largely written round Dr. Haig's own particular theories, these theories are not obtrusively put forward. The value of the teachings of this book must, too, be determined by their practical results, and not by any academic or physiological controversies.

A Handbook of Hygiene and Sanitary Science. By George Wilson, M.A., M.D., LL.D. Edin., F.R.S. Edin., D.P.H. Camb. Eighth edition. (London: J. and A. Churchill. 1898. Price 12s. 6d. Pp. 798.)

An eighth edition does not require praise. The steady demand which calls for one issue after another is sufficient proof that the matter and the arrangement of the book have secured for themselves the approbation of the public to which it is addressed. Yet there are dangers in popularity, for hygiene is a most progressive branch of knowledge, and a writer who has found his work so well received as Dr. Wilson has been, might well feel tempted to rest on his oars and bask in the success which he has achieved. One's first thought, then, in reviewing such a work is to inquire whether it is merely a reprint or has been brought properly up to date. In regard to this there can be no doubt; everywhere there are signs that the author has kept himself in touch with all that is going on, and has judiciously availed himself of recent sources of information in the revision of his book. Thus we find references to recent outbreaks of milk-borne typhoid and of ptomaine poisoning, and to the transmission of typhoid fever by oysters and other forms of shell-fish. Much has been added on the treatment of house refuse and sewage, a full description being given under the latter heading of the various biological processes which are now coming so much into vogue.

Under the heading "Hospitals" reference is made to several quite recent examples of hospital construction. In regard to isolation hospitals an important matter is touched upon, namely, the question of sites. It is pointed out that "the difficulties in respect to sites for the isolation of cases of small-pox, as well as of other cases of infectious disease, are greatly increased by the conditions laid down by the Local Government Board," and it is added that the "Local Government Board are so impressed with the dangers of aerial connection to long and undefined distances that no loans can be granted for the extension of an isolation hospital, or the erection of any new hospital, unless an undertaking is entered into that no building or block within the . . . prescribed limits shall be used for the reception of cases of small-pox. This practically means that local authorities . . . must procure two hospital sites," and the author attributes a good deal of the lack of provision for small-pox which is manifest on every side to "the difficulties of obtaining suitable dual sites under the conditions laid down by the Local Government Board."

Under the head of "Infectious and Parasitic Disease" a fairly full description is given of modern methods and theories, but it is in this chapter that Dr. Wilson more especially shows his individuality, for he sets himself in opposition to a great deal which it is now the fashion for sanitarians to accept without question in regard to infection, immunity, protective inoculation, and serum therapeutics. While, however, we may admit to the full the force of some of Dr. Wilson's criticisms of modern theories, and quite agree with him when he says that "the trend of microbial investigation has of late years threatened to ignore or minimise the influence of those conditions of environment without which many pathogenic organisms are powerless for mischief," yet we cannot go with him in his general scepticism as to bacteriological results. Still it is always well that someone should raise his voice against a too great receptiveness towards new doctrines and a too ready subserviency to popular and fashionable creeds. There is a careful chapter on "Disinfection," and then follow valuable and well-written chapters on "Vital Statistics," on "Sanitary Law," and on "Sanitary Officers and Their Duties," which will be found very useful. It is to be noted that while this work will serve well as a text-book to the general student, the main purpose for which the book was first written was that it should serve as a guide to medical officers of health and other sanitary officers, as well as a practical work of reference for general practitioners who are interested in sanitary progress, and there can be no doubt that in its new and improved form the book still serves this purpose admirably well.

